# Further Remarks on the Iodide of Potassium in Pneumonia

**Published:** 1845-06

**Authors:** George L. Upshur

**Affiliations:** Norfolk, Virginia


### MEDICAL EXAMINER,

AND

RECORD OF MEDICAL SCIENCE.

NEW SERIES—No. VI.—JUNE, 1845.

### ORIGINAL COMMUNICATIONS.

### *Further Remarks on the Iodide of Potassium in Pneumonia.*

By GEORGE L. UPSHUR, M. D., of Norfolk, Virginia.

To the Editor of the Medical Examiner.

In the 13th number of the Examiner for 1844, I published some observations upon the use of the iodide of potassium in the suppurative stage of pneumonia. A few days since I received the March number of the *Journal des Connaissances Médico-Chirurgicales*, published at Paris by MM. I. Lebaudy, H. Gouraud and Martin Sauzer, in which I find the following comments upon the article above referred to:

"Although iodine has gradually invaded the entire therapeutic field of chronic diseases, it is a matter of surprise to see it entering into that of acute affections, especially of such a one as pneumonia. It is the more wonderful that it is recommended in the *suppurative period* of this disease. We say, however, that the case reported by the author is not sufficiently in detail; he there merely states that the sputa lost their rusty appearance to assume an aspect more decidedly purulent." * * * * * "Since the month of January, the author has employed the iodide of potassium in six other cases of pneumonia, in neither of which did the treatment disappoint his expectations. We regret that M. Upshur has not thought proper to insert these seven cases in the *Medical Examiner*, instead of trusting solely to one simple observation. Certainly the subject is well worth the trouble. Without suspecting the good faith of the author, the fact which he maintains is so very extraordinary, that few practitioners will give credence to it, unless he brings further proof upon the subject."

The notes of three of these cases have unfortunately been mislaid; the fourth has already appeared in the Medical Examiner for June 29th, 1844, and I desire now to place a detailed account of the remaining three in the columns of that Journal. I am prompted to this solely by the last clause in the extract just quoted, for my own mind has long since been convinced of the propriety of the treatment recommended.

Case I.—John Fisher, a lad aged 16, of a delicate frame and lymphatic temperament—previously healthy—after exposure to a chilling rain, was seized on the evening of January 8th, 1844, with severe rigors, which were followed by high febrile action, with a hacking cough and a sense of tightness across the chest. I saw him about noon the next day, when his condition was as follows: Passed a restless night; feels much debilitated; lies upon his back, with the shoulders raised; respiration 45 per minute, but performed without pain; cheeks flushed, and nostrils dilated; pulse 120, soft and full; skin hot and dry; bowels disposed to looseness; cough very troublesome, and expectoration consists of tenacious mucus; much thirst; appetite impaired, and countenance anxious. Physical examination of the chest gave dull percussion over the lower lobe of the right lung posteriorly, with very distinct crepitant rhonchus and an expiratory sound. The percussion over the left lung was clear, and the respiration merely puerile.

*Prescription.* Cut cups posteriorly to the right lung; and one of the following powders every hour: ℞, Calomel. gr. x; Pulv. Ipecac. gr. v. M. Div. in chartulas viii.

Jan. 10.—More dyspnœa; inclined to sleep, but is prevented by the cough; expectoration to-day exhibits some rusty streaks; respiration 47 per minute; pulse 112, and jerking; tongue dry, and pointed with a dark coating posteriorly; not as much thirst as yesterday; bowels were acted on four times during the night; complains of no pain, but when questioned, merely answers that he "feels very sick." Physical signs as yesterday, except that the expiratory sound in the lower lobe of the right lung is more distinct. Prescribed tart. emetic. gr. ij. tr. opii camph. f.ʒij. Aquæ f.℥jv. M. A table spoonful every two hours. In the evening there was an exacerbation of fever, and the solution having excited vomiting was given in half the original dose.

On the 11th, the patient's condition was little altered. The tartar emetic was disposed to run off by the bowels, and therefore was discontinued for the present, and the mild chloride of mercury, in doses of one grain every hour, was substituted in its stead. The sputa were more *rusty*, and in the diseased lung the crepitant rhonchus had almost entirely disappeared, while the blowing respiration was heard very distinctly.

During the five days following, the condition of the patient was not materially changed, and the treatment consisted in the cautious administration of the tartar emetic, alternated with mercurials. On the 17th, the pulse was only 103 per minute, and extremely feeble; the respiration 30, and performed with difficulty; the cough troublesome, and expectoration, consisting of pus, sometimes marked with bloody striæ, was very abundant. In addition to the dull percussion and bronchial respiration, there was now and then heard a crackling sound, as if the air was forced through a tenacious fluid. *Prescription.* Emp. Canth. 4 in. by 6 in. posteriorly to the right lung. ℞. Potassii iodidi ℈i. Infus. humuli f.℥viij. M. A wine-glassful every eight hours.

On the 18th, he was yet more feeble; pulse as yesterday, but with less volume; tongue dry, and covered with a brown crust; blister, which remained on eight hours, drew but little; dyspnœa so great as to oblige him to be propped up with pillows; bowels very loose, the evacuations being watery. Ordered wine-whey. Tincture of kino in doses of thirty drops, every half hour, until the alvine evacuations ceased: continue iodide of potassium.

16th—Rested well last night; cough troublesome only at times; feels stronger, and desires something to eat; pulse 95, soft, and fuller than yesterday; respiration 25, but accompanied with some dyspnœa; one evacuation from the bowels during the night; percussion still very dull, and respiration bronchial, with harsh submucous rhonchus. Took about two ounces of wine-whey, and one and a half drachm of the tincture of kino. Continue the iodide of potassium in doses of gr. v. every six hours, administered in f.℥ij. of infusion of hops.

On the 20th, the patient was decidedly better, and, it might be said, was upon the verge of convalescence. On the 21st, private business called me from the city, and I requested my friend, Dr. Wm. I. Moore, to attend to the case for me. He continued the use of the iodide of potassium in the same doses as I had prescribed it, and the patient convalesced so rapidly, that he considered further attendance useless after paying the fifth visit.

Case II.—Mrs. M—— aged 30, the mother of three children, of a vigorous constitution, was taken ill on the 22d of February, 1844, with ague, followed by fever. Thinking her disease only a slight catarrh, she did not send for me until the 26th, when I found her labouring under high febrile excitement, cough and dyspnœa. Upon examining the chest, I detected crepitant rhonchus diffused through the lower lobe of the left lung. The percussion there was but little duller than natural, and the expiratory sound scarcely discernible. Her bowels were constipated; skin moist; anorexia complete; expectoration very scanty; some headache; reclines upon the back. The pulse was 110 per minute, small and compressible; the respiratory act was so interrupted by the cough, that the number of respirations per minute could not be accurately determined: the average, however, amounted to about 35. Ordered cut cups to the left side posteriorly, and one of the following pills every hour: ℞. Calomel. gr. x; tart. emet. gr. iss; syr. simp. q. s. M. Div. in pil. viii.

27th. No material change. 28th. On this day the patient became worse, complaining of a heaviness in the præcordial region, and difficulty of drawing the breath. The action of the heart was confused and labored, giving to the pulse at the wrist an intermittent character. Upon applying the ear to the breast, an intense "bellows sound" was heard. The respiration in the diseased portion of the left lung was decidedly bronchial, and the percussion flat. It will be perceived from these symptoms that the pneumonia had passed from the first into the second stage, and also had become complicated with endocarditis, a complication not uncommon in frank pneumonia, and the intensity of which often masks entirely the original disease. *Prescription*. Cups to the præcordia. ℞. Potass. nitrat. gr. xl.; tinct. digitalis gtt. xlv.; aquæ f.℥jv. M. A table-spoonful every two hours.

This mixture was exhibited, with now and then an intermission, until March 2d, when it was entirely discontinued, and cut cups were again applied to the region of the heart. The action of the heart was more regular than on the day the cups were first applied, but the "bellows sound" still existed. There was no alteration in the inflamed lung, though the dyspnœa was not so urgent, nor the cough so harassing. The sputa were transparent, and so viscid that they would cling to the bottom of the vessel when it was turned down. Pulse 100, feeble; mouth dry, and tongue coated with a dark fur; patient somewhat deaf and very drowsy; bowels constipated. Blisters applied to the ankles, and one drop of oleum tiglii given internally. March 3. Croton oil acted copiously, and the cantharides plasters vesicated well, which relieved the drowsiness entirely, and patient says she feels more comfortable than usual. On the 4th there was a sudden change in her condition. She was extremely feeble; pulse soft, irritable, almost fluttering; respiration obstructed, and in the left lung, lower lobe, a harsh mucous rhonchus accompanying the bronchial respiration; cough very frequent, and expectoration abundantly purulent. I ordered a decoction of senega and infusion of serpentaria combined, and exhibited in doses of a wine-glassful every hour. Under this treatment the patient rallied, and continued to expectorate with considerable ease until about noon on the 5th, when the influence of the remedy seemed to be lost, and the depression of the vital powers was again approaching. Wine whey was now exhibited, but it only accelerated the pulse without adding to its volume or increasing the patient's strength. As a dernier ressort, I now prescribed the iodide of potassium, in the same dose and in the same manner as prescribed in the case of John Fisher, detailed above. In twenty-four hours from this time there was an evident improvement in the condition of the patient. She was stronger; the pulse was fuller and stronger, and the expectoration much more easily performed. This treatment was continued, and the appetite returning in three or four days, a nourishing diet was directed to be taken, and the patient was perfectly well on the 17th of March, with the exception of slight cough, and a roughness of the first sound of the heart. The roughness still exists, after the lapse of more than a year, and as it is probably dependant upon thickening of the aortic semilunar valves, it may remain for some time to come. The patient, however, in other respects has enjoyed perfect health up to this time.

Case III.—The subject of this case is Mrs. B——, wife of the sexton of St. Paul's Church. She was taken sick about the middle of April, 1844, but owing to my absence from the city at that time, I did not see her until the 25th. From the account she gave of her situation, she had been labouring under pneumonia of the lower lobe of the right lung, which was ushered in by the usual symptoms of chill, fever, cough and oppression of respiration. She had taken only a cathartic of senna and manna, and drunk copiously of flaxseed tea. The following were her symptoms at my first visit: Pulse 95, and feeble; respiration somewhat difficult; expectoration abundant, and consists of pus floating in a glairy mucus; no appetite; bowels opened regularly once a day. *Physical signs.*—Percussion clear throughout both lungs, except over the lower lobe of the right, where it is perfectly flat. Respiration over this lobe intensely bronchial, with crackling and submucous rhonchus. I ordered a blister to the diseased portion of the right lung, and the iodide of potassium internally, as I had heretofore prescribed it. It is useless to pursue the details of the case further. Suffice it to say, that every symptom of disease was rapidly ameliorated, and the patient in ten days returned to her household duties. She has been perfectly well since that time.

*Remarks.* As stated in my first communication on this subject, I do not attempt to explain the *rationale* of this mode of treating the suppurative stage of pneumonia. It suggested itself, in the first instance, when every other treatment had proved abortive, and its prompt beneficial effect, in that case, recommended it in the management of similar cases which subsequently came under my notice. Iodine is certainly more rapidly alterative than any other therapeutic agent, and in addition to its alterative action, the experiments of Jörg, according to Dr. Dunglison, go to establish the fact that it produces a decidedly stimulant effect upon the respiratory organs. This stimulant impression is much needed in the suppurative stage of pneumonia—a period which is usually characterized by subsidence of inflammatory action, and great debility of the respiratory function. Lugol and Eager both observed, that the appetite and nutrition rapidly improved under the use of iodine and its preparations, an effect which is greatly to be desired towards the termination of so acute an affection as pneumonia. To conclude: if I were disposed to hazard a conjecture upon the *ratio medendi* of the iodide of potassium when administered in the third stage of pneumonia, I should say: 1. That it tends to promote the absorption of the coagulable lymph effused into the parenchyma of the lungs during the second stage, the breaking down of which lymph into pus serves to keep up the harassing cough and hectic irritability of the third stage, both of which are so often prolonged for a considerable time after all inflammatory action has ceased. 2. That it is directly stimulant to the lungs at a time when they most need a stimulus. And 3, that it invigorates the digestion and promotes nutrition, thereby rapidly restoring the enfeebled frame to the normal condition of health.

*Norfolk, (Va.,)* May 10th, 1845.

